# The stable isotope composition of nitrogen and carbon and elemental contents in modern and fossil seabird guano from Northern Chile – Marine sources and diagenetic effects

**DOI:** 10.1371/journal.pone.0179440

**Published:** 2017-06-08

**Authors:** Friedrich Lucassen, Wolfgang Pritzkow, Martin Rosner, Fernando Sepúlveda, Paulina Vásquez, Hans Wilke, Simone A. Kasemann

**Affiliations:** 1 Department of Geosciences and MARUM—Center for Marine Environmental Sciences, Universität Bremen, Bremen, Germany; 2 Department of Analytical Chemistry and Reference Materials, Bundesanstalt für Materialforschung und -prüfung (BAM), Berlin, Germany; 3 IsoAnalysis UG, Berlin, Germany; 4 Geología Regional, Servicio Nacional de Geología y Minería, Santiago de Chile, Chile; 5 Departamento de Geología, Universidad Católica del Norte, Antofagasta, Chile; University of Otago, NEW ZEALAND

## Abstract

Seabird excrements (guano) have been preserved in the arid climate of Northern Chile since at least the Pliocene. The deposits of marine organic material in coastal areas potentially open a window into the present and past composition of the coastal ocean and its food web. We use the stable isotope composition of nitrogen and carbon as well as element contents to compare the principal prey of the birds, the Peruvian anchovy, with the composition of modern guano. We also investigate the impact of diagenetic changes on the isotopic composition and elemental contents of the pure ornithogenic sediments, starting with modern stratified deposits and extending to fossil guano. Where possible, ^14^C systematics is used for age information. The nitrogen and carbon isotopic composition of the marine prey (Peruvian anchovy) of the birds is complex as it shows strong systematic variations with latitude. The detailed study of a modern profile that represents a few years of guano deposition up to present reveals systematic changes in nitrogen and carbon isotopic composition towards heavier values that increase with age, i.e. depth. Only the uppermost, youngest layers of modern guano show compositional affinity to the prey of the birds. In the profile, the simultaneous loss of nitrogen and carbon occurs by degassing, and non-volatile elements like phosphorous and calcium are passively enriched in the residual guano. Fossil guano deposits are very low in nitrogen and low in carbon contents, and show very heavy nitrogen isotopic compositions. One result of the study is that the use of guano for tracing nitrogen and carbon isotopic and elemental composition in the marine food web of the birds is restricted to fresh material. Despite systematic changes during diagenesis, there is little promise to retrieve reliable values of marine nitrogen and carbon signatures from older guano. However, the changes in isotopic composition from primary marine nitrogen isotopic signatures towards very heavy values generate a compositionally unique material. These compositions trace the presence of guano in natural ecosystems and its use as fertilizer in present and past agriculture.

## Introduction

Nitrogen and carbon are present in many natural organic compounds, and stable isotope compositions of nitrogen (N) and carbon (C) are widely used in studies of ecology in marine and terrestrial environments [1]. Each element has two stable isotopes, ^15^N and ^14^N and ^13^C and ^12^C, respectively. Isotope ratios of ^15^N/^14^N and ^13^C/^12^C are commonly reported as δ^15^N and δ^13^C relative to a reference value that is the N isotopic composition of air and C isotopic composition of the Vienna Pee Dee Belemnite ($\delta sample=\left(\frac{isotope\;ratio\;sample}{istope\;ratio\;standard}-1\right)\;*\;1000\;[‰]$). Both isotope systems show fractionation [1, 2] between the respective high and low mass isotopes during formation and decomposition of organic materials. The stable isotope composition changes systematically in terrestrial and marine food webs from producers to higher trophic (feeding) levels, with a general increase in δ^15^N and δ^13^C in the organism along ascending food chains [3–6]. The systematic variation in δ^15^N along the marine food chain is +3 ±1 ‰ for each trophic level [3, 7, 8]. Furthermore, the marine nitrogen cycle generates regionally variable compositions of δ^15^N in the nutrients [9, 10] and causes regional offsets in the systematic variation of δ^15^N along trophic chains from producers to vertebrates [7, 11–13]. Terrestrial and marine ecological systems show differences in the δ^15^N signatures of the producers with ^15^N enriched in marine producers [14]. The high trophic levels of seabirds depend on their specialized feeding, e.g. of certain species of euphausiidae or fish [12]. The feeding levels of birds can be determined by analyses of δ^15^N, e.g. in faeces (guano in the following) [15]. Seabird guano transfers considerable amounts of N and other nutrients into coastal terrestrial ecosystems and connects marine and terrestrial food webs [16, 17]. The heavier δ^15^N marine signature is used as a tracer for the input of marine materials in modern ecosystems and also as an indicator of changes in the birds’ diet, i.e. changes in marine biological productivity in fossil settings [18–23].

Most studies on natural ecosystems investigate the influence of guano on soils with a focus on nutrient supply and cycling in humid climates, where the weathering of silicate minerals, the activity of plants, and the mobility of material in aqueous solutions are important processes in ornithogenic soil formation [18–23]. In arid climates, guano forms the main constituent of ornithogenic sediments through different stages of decomposition (diagenesis) and represents a relatively simple compositional system. Large amounts of seabird guano were accumulated at the Pacific margin in Peru and Northern Chile [24, 25] during the longstanding (hyper)arid climate conditions, since at least the late Miocene [26–30]. Historically, the large scale extraction of the Peruvian and smaller Chilean deposits for exportation as fertilizer to Europe and the USA occurred to the point of exhaustion of these fossil deposits during the 19th century [31]. The collection of guano as fertilizer is still of local importance [32]. However, guano was already used as a fertilizer by pre-Hispanic Andean communities [33, 34]. Archaeological studies of man-made ecosystems in agriculture focused on the impact of guano as fertilizer on the foods’ stable isotope composition and hence the consumers, i.e. humans and domestic animals of pre-Hispanic communities in Peru and Chile [33–35].

The present producers of guano deposits in Northern Chile and Peru are a few species of specialized colonial seabirds (so called guano-birds; [25, 36, 37]) that restrict their exclusively marine alimentation to small, non-migratory schooling fish. Their preferred prey is the Peruvian anchovy (*Engraulis ringens*), which is endemic to the coastal upwelling zone of Chile and Peru between approximately 6° and 42° S [38], and sardines (*Sardinops sagax*). Anchovies and other potential prey feed on zoo- and phytoplankton, which is produced in the nutrient-rich waters of the upwelling zone off the Pacific margin [39, 40]. The position of the guano-birds in the trophic chain is well defined by their selective alimentation, and a pure marine signal is expected in the birds and their excreta [12].

**Fig 1:**
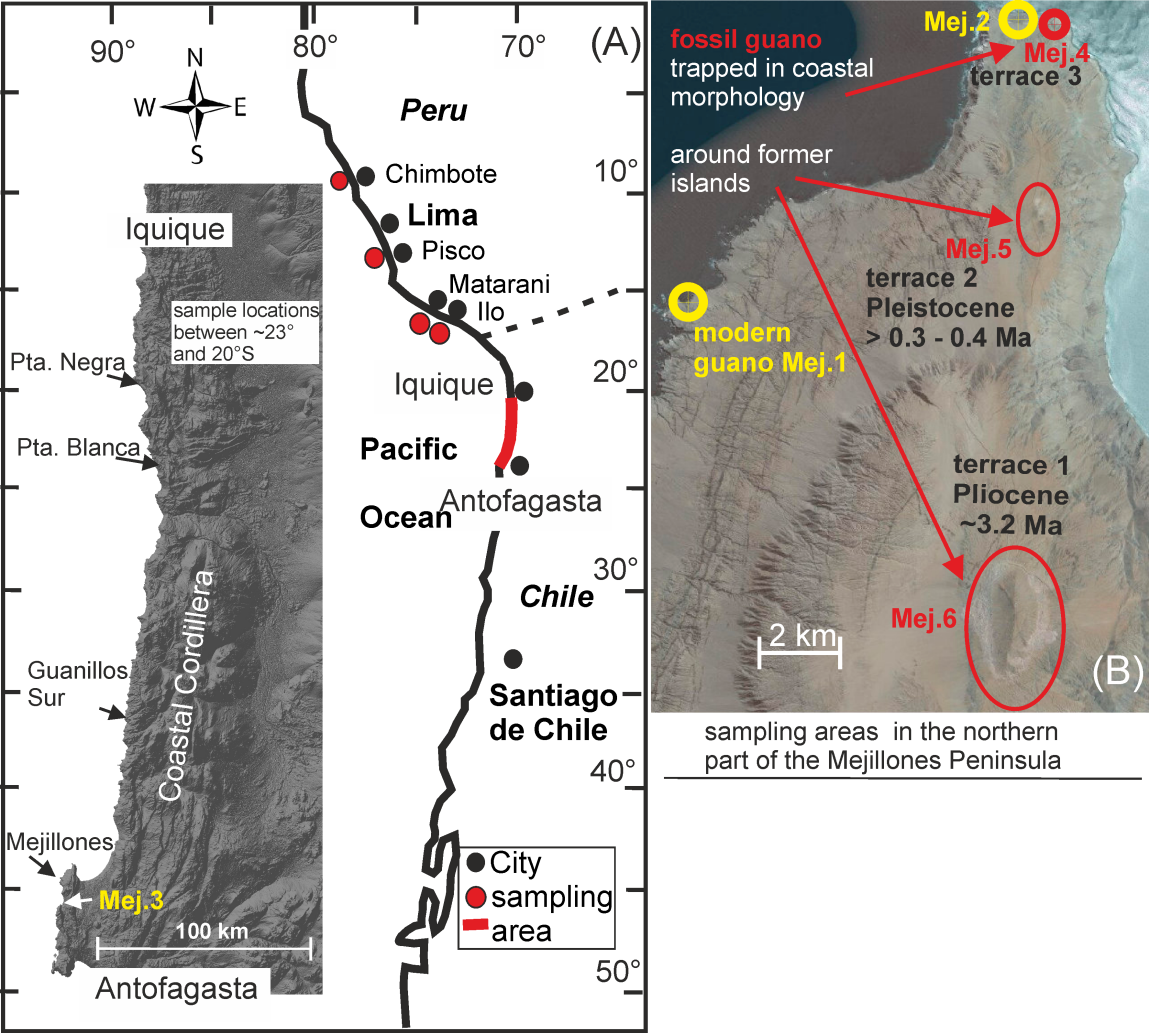
Sample locations and examples of sample sites. (A) Overview of the sample area in Northern Chile between Antofagasta and Iquique (red line) and sample areas of fish (red dots) off Peru (Chimbote, Pisco, Matarani, Ilo are the locations of the respective ports and fishmeal plants). The satellite view [44] covers the sampling area in Northern Chile with our sample locations north of the Mejillones Peninsula and the position of sample location Mej.3: The mountain chain with the steep escarpment to the Pacific Ocean is the Coastal Cordillera. (B) The enlarged northern part of the Mejillones Peninsula [45] shows sample locations of modern guano (Mej.1, Mej.2) and fossil guano (Mej.4) *in-situ* profiles. Guano at Mej.5 (Cerros Tetas) and Mej.6 (Morro Mejillones) is resedimented around former islands that rest on fossil marine terraces.

The knowledge about guano deposits in Northern Chile is restricted to numerous studies from the 19^th^ and early 20^th^ century that describe the geology of the deposits and the major element composition of the guano, both of which are related to the former economic importance of guano (summary in [25]). The potential of modern guano and fossil deposits for the study of marine signatures has not yet been fully explored. In the present pilot project, we revisited Recent to Pliocene guano deposits at the Pacific coast of Northern Chile (Fig 1A) and sampled stratified deposits from active and fossil deposition sites. We analysed C and N isotopic composition and elemental composition in fresh guano, as well as during guano decomposition and diagenesis. A strong systematic variation in N isotopic composition to heavier δ^15^N (and heavier δ^13^C in time series of modern guano) and the passive enrichment of non-volatile elements during the loss of N and C are the main effects of diagenesis in the guano.

We also analysed N and C isotopic composition in modern Peruvian anchovy fishmeal from catches off Peru (Fig 1A). The isotopic composition of the fishmeal specifies the marine baseline at the birds’ feeding level. Surprisingly, N and C isotopic compositions revealed systematic latitudinal changes along the Peruvian coast between 9° to 17° S. This variation can be extrapolated towards the South into our study area in Northern Chile by using published δ^15^N data from surface sediments below the upwelling zone in Peru and Chile [41–43].

We find that the strong effect of diagenesis on the δ^15^N and δ^13^C values in fossil guano complicates or prevents their use in ecological studies, e.g. in reconstructing the feeding levels of birds or detecting changes in marine δ^15^N and δ^13^C values. On the other hand, it’s nearly unique heavy δ^15^N signature makes guano an excellent tracer of marine input into natural and man-made terrestrial ecosystems.

## Guano deposits and fishmeal: Geology, age, and sampling strategy

Guano birds prefer nesting and resting realms such as small islands, cliffs, and beach scarps, all of which are at the coastline and are inaccessible for onshore predators. Consequently, deposits of seabird guano are found directly at or close to the coastline (Fig 1A and 1B). Large parts of the coastal area in Northern Chile are under variably strong uplift, which together with sea-level changes led to the formation of terraces during the late Cenozoic [46–52]. Locally, former islands (now hills) rest on these terraces as in the northern part of the Mejillones Peninsula (Fig 1B; [52, 53]. Therefore, modern guano deposits are found directly at the coastline, while fossil guano deposits also occur away from the modern habitat of the birds.

**Fig 2:**
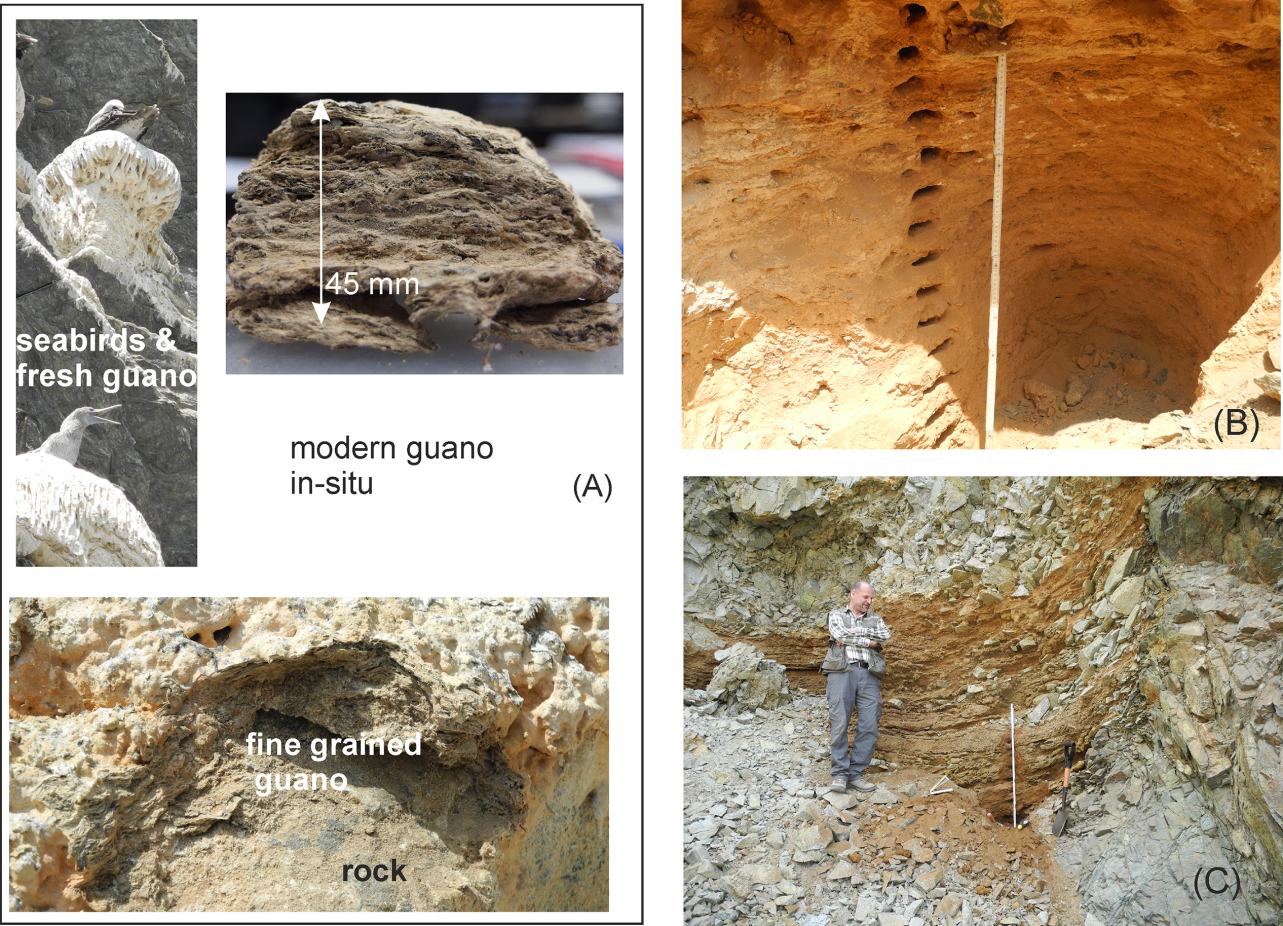
Occurrence of guano in the field. (A) Modern guano at location Mej.1 with some of the primary guano producers and the situation below the nesting place. Laminated solidified guano (45 mm profile; upper right image in the box) rests upon a layer of unsolidified fine-grained guano on the bedrock (image at the bottom of the box). (B) Fossil guano of the *in-situ* deposit Punta Blanca. (C) Fossil guano, hill slope sediment at Mej.5: reddish guano is intermingled with local rock debris.

An extended description [24] of all major North Chilean guano occurrences distinguishes between *in-situ* deposits and resedimented guano. *In-situ* deposits are restricted to traps in the coastal morphology like gullies, caves, and fractures with fossil and modern formations at resting areas or nesting places (Fig 2A and 2B. Resedimented guano is exposed around former islands or at the foot of cliffs. The guano in hill-slope sediments is intercalated with boulder and stratified rock detritus from local sources (Fig 2C). All guano deposits listed by [24] have been heavily mined at an industrial- or artisanal scale, and many have been exploited to exhaustion. Rare ongoing mining activities are artisanal according to our own observations.

The description of W. Biese [24] was used as a guide to the historical guaneras (guano mines). Locations for collection of modern guano were selected using satellite images. Our sampling strategy considered that precise age data on guano are not available and that the potential for dating beyond the limit of the ^14^C method at ca 50 ka is hitherto unexplored. Therefore, one focus of our field work was the northern part of the Mejillones Peninsula. Here, age relations between the occurrences of fossil guano are roughly known from the dating of the terraces, which host the former islands (Fig 1B). We distinguished modern guano, where deposition is ongoing or recent, from fossil guano, which is located off the birds’ present habitat based on field evidence. Selected locations with active deposition were dated by ^14^C (Table 1). The stratigraphic columns in fossil guano (1 to 4 m thickness) were sampled in 5 to 20 cm intervals. Each sample presents approximately 1 cm of sediment collected parallel to the bedding plane (Fig 2B). Modern guano was sampled at a much finer scale in <1 cm to 1–2 mm intervals parallel to the bedding plane (profile Mej.1; Fig 2A). The sample size is between 20 to 40 g in fossil guano and a few grams at fine scale sampling of layered modern guano. The coordinates of sample locations, the spacing of sample profiles, and the respective depths below surface are given in S1 Table.

**Table 1:** ^14^C systematics and modelling.

Location/sample	depth below surface	^14^C age BP or pMC*** (percent Modern C)	^14^C calendar age in years; marine13 and ΔR 200 ± 140 *; 1950 = 0 y	^14^C calendar age in years; atmosphere SHCal13**; 1950 = 0 y
**Mejillones 1 nesting place** S23° 03' 59" W70° 33' 25"				
Mej.1-123-14	43 mm	103.95 ± 0.34 pMC		
Mej1-129-14	50 mm	104.32 ± 0.31 pMC		
**Mejillones 2 resting place** S23° 01' 38" W70° 30' 37"				
Mej2-110-14-2	7.5 mm	380 ± 30 BP	1860 ± 50 AD	1550 ± 50 AD
Mej.2-107-14	36 mm	1060 ± 40 BP	1490 ± 130 AD	1040 ± 55 AD
Mej2-105-14	55 mm	1230 ± 40 BP	1180 ± 50 AD	850 ± 65 AD
**Mejillones 3 resting place** S23° 04' 52" W70° 34' 09"				
Mej.3-80-14	15 mm	104.82 ± 0.35 pMC		
Mej.3-81-14	15 mm	105.29 ± 0.35 pMC		
**Punta Negra** S20° 50' 10" W70° 10' 44"				
PtaN-153-13	20 mm	1095 ± 30 BP	1460 ± 125 AD	990 ± 35 AD
PtaN-157-13	320 mm	2990 ± 60 BP	565 ± 180 BC	295 ± 75 BC
**Fishmeal**				
11 Chimbote S9°		102.23 ± 0.37 pMC		
15 Pisco S13°46“		102.32 ± 0.33 pMC		
4 Ilo S17°38“		100.99 ± 0.44 pMC		

* ΔR calculated from 6 local values [74], marine13 [70]

** atmosphere curve southern hemisphere SHCal13 [71]

*** conversion factor to F^14^C notation = 0.01

Modern to fossil guano is exposed at the Mejillones Peninsula (Fig 1B). Sample sites Mej.2, Mej.4, Mej.5, and Mej.6 rest on tonalite, Mej.1, Mej.1a on late Palaeozoic metasediments, and Mej.3 on Jurassic mafic intrusive rocks (geology of the Mejillones Peninsula in [54]). All guano deposits north of the Mejillones Peninsula were considered as fossil in the field (Fig 1A) and rest on Jurassic intrusive and volcanic rocks.

Eighteen commercial fishmeal samples were provided by Köster Marine Proteins GmbH, Hamburg via the Terminal managed by J. Müller Weser, Bremen, Germany. These samples can be traced back to the ports of origin, which also host the fishmeal producers according to the loading documents. The anchovy-fishery is local by purse seine boats that operate at a maximum distance of approximately 60 km offshore with short trip durations rarely exceeding 24 to 30 h [55, 56]. Therefore, the catch can be attributed without large deviations to the latitude of the port (Fig 1A; S2 Table).

### Mejillones Peninsula modern guano

Profile Mej.1 of the *in-situ* type is located at 1.5 m above sea level (a.s.l.) beneath occupied nesting places of the Peruvian Booby (*Sula variegata*) [57]. The profile comprises 45 mm of finely-laminated solid white guano and approximately 10 mm of soft granular guano at the contact to the underlying solid metasedimentary bedrock (Fig 2A). Erosion features are absent despite the potentially discontinuous deposition of guano inferred from the seasonal occupation of the nests. The fine layering of <1 mm is caused by the deposition of fluid excreta and almost instantaneous drying. The uppermost guano-layers are recent (collected in 2014 AD).

The position of this deposit is in a small bay that is shielded from the waves of the open Pacific by a cliff. Water can only reach the deposit with exceptionally high waves.

Profile Mej.2 of the *in-situ* type is located at 110 m a.s.l. at the very edge of the coastal escarpment (Fig 1B). It represents a deposit at an active resting place. The base of the 75 mm thick deposit is formed by small, rounded shell fragments and sand that may present late Pleistocene beach sediments of terrace 3 [52] deposited on tonalite.

The group “other modern guano” comprises surface samples from three distant areas. We sampled active resting places on the Mejillones Peninsula at 20 m a.s.l (Mej.1a near profile Mej.1; Fig 1B) and a small rocky promontory 15 m a.s.l at Mej.3 (20 km south of Mej.1, Fig 1A), and a third location off the Mejillones Peninsula, south of Antofagasta at the edge of the coastal escarpment (samples G-6-14, G-7-14, G-8-14, G-9-14; S1 Table). The aim of the random collection was to control the compositional variability of observational modern guano.

### Mejillones Peninsula fossil guano

Profile Mej.4 is located at 135 m a.s.l. on terrace 3 (Fig 1B) and comprises 4 m of guano of the *in-situ* type. The deposit has a late Pleistocene maximum age according to the subdivision of terraces [52].

The resedimented deposit Mej.5 is located at 310 m a.s.l. in the hill slope and footwall around Cerros Tetas (Fig 1B) and rests on the oldest Pleistocene terrace 2 of 400–300 ka according to ^10^Be and ^21^Ne surface dating (Figs 1B and 2C; [52, 58]).

The resedimented deposit Mej.6 is located at 550 to 580 m a.s.l. in the footwall of the hill slope along the eastern section of Morro Mejillones (Fig 1B) and rests on the oldest Pliocene terrace with an age of 3.2–2.8 Ma [52, 59, 60].

### Guano deposits north of the Mejillones Peninsula

These deposits are described in the order of their occurrence towards the North (Fig 1A): The profile Guanillos sur is located at 50 m a.s.l. and comprises 350 cm of stratified guano of the *in-situ* type, which fills the morphology of Jurassic volcanic rocks. The five samples that were chosen for analyses are virtually free of rock fragments. Guanillos shares the same tectonic block with terraces north of the Mejillones Peninsula (200–300 ka; [47, 51]), hence a maximum late Pleistocene age can be inferred.

The profile Punta Blanca is of the *in-situ* type and located at 20 m a.s.l. in an active artisanal mine. The 100 cm thick stratified guano was deposited in a morphological trap (Fig 2B). A maximum late Pleistocene age is inferred from the terrace (approximately 120 ka from the nearby location Guanillos del Norte [49]).

The resedimented deposit Punta Negra is located at 10 m a.s.l. The 32 cm of guano are deposited from surrounding boulders (resting places) into a shallow gully. The terrace indicates a maximum age of approximately 100 ka [48, 49].

## Materials and methods

### Sample mineralogy and preparation

The fresh metabolic waste of birds consists of > 50–80% uric acid, minor amounts of urea, and ammonia [61–63] and variable but small proportions of undigested remains of food, fish in the case of seabirds.

Crystalline organic compounds in modern and fossil guano are mainly water-insoluble uric acid, different crystalline phases of the phosphate and oxalate groups, and salts mainly halite from seawater and sal ammoniac [25]. Guano deposits are furthermore a source of rare minerals described in the mineralogical literature ([64, 65] and references therein). We surveyed crystalline phases on selected powdered samples by XRD (X-ray Diffraction), using a Bruker D2 Phaser and PANanalyticals’ HighScore evaluation software at Technische Universität Berlin, FG Mineralogie. The detection limit was 1–2 volume % of the respective mineral. Modern guano consists of uricite (uric acid; C_5_H_4_N_4_O_3_) and minor salts of the uric acid (urates), the oxalate minerals whewellite Ca(C_2_O_4_) H_2_O and rarely weddellite Ca(C_2_O_4_) 2H_2_O, the calcium phosphates whitlockite Ca_9_(Mg,Fe)(PO_4_)_6_PO_3_OH and minor (hydroxyl)apatite Ca_5_(PO_4_)_3_(F,Cl,OH), additional halite (NaCl), and sal ammoniac (NH_4_Cl). Fossil guano comprises mainly whitlockite and (hydroxyl)apatite, calcium oxalate, and rarely some uricite. Secondary minerals unrelated to the guano are rare gypsum in fossil guano that is a typical authigenic mineral in the soils of arid climate zones and halite. The latter precipitated from seawater and forms the principal cement especially in modern guano. The seawater is transported by birds and sea spray.

Other secondary components in guano samples originate from the depositional environment and comprise clastic materials. Organic clastic materials include mostly feathers and occasionally bones from birds and fish. At a few locations, samples contain minor detrital carbonate from shell fragments. A detailed screening of organic clastic components in fossil guano from the lower Pleistocene terrace 2 (our location Mej.5) of the Mejillones Peninsula indicates the preservation of bird and fish remains [58] including still existing species, namely the Peruvian diving petrel (*Pelecanoides garnotii*) and the Peruvian anchoveta (*Engraulis ringens*). Resedimented guano generally contains rock fragments and/or silicate minerals from the respective hill-slope.

Sample treatment follows the above observations of various components in the guano. Clastic components were removed by hand picking under the stereo microscope and by sieving before homogenizing the sample in an agate mortar. This was the only treatment applied to samples labelled “untreated”.

An aliquot of the untreated sample was weighed into centrifuge tubes and subjected to leaching with 18.2 MΩ water in order to remove the water soluble fraction. The leached samples are labelled as “treated”. The leaching procedure included three washings, centrifuging between the washings, and collecting of the supernatant after each washing. Finally, the treated samples were dried at 80° C and reweighed. The water soluble fraction crystallizes dominantly halite (NaCl) after evaporation, but in modern guano small amounts of urea and ammonium (NH_4_)-, nitrite (NO_2_)-, and nitrate (NO_3_) salts are also present [25]. We analysed both treated and untreated samples in order to distinguish the influence of potential water-soluble N-bearing salts on the isotopic composition. The contents of the water soluble fraction range from 20 to 50 wt% in samples classified as modern guano in the field, but are generally below 20 wt% in fossil guano (S1 Table). Rare samples with carbonate detritus were leached in a second step in 1 molar acetic acid until CO_2_ formation ceased, followed by three washings with 18.2 MΩ water.

### Analytical methods

Carbon and nitrogen isotope ratios and elemental concentrations were analysed with an elemental analyser (Vario EL III) coupled with an isotope ratio mass spectrometer (IRMS IsoPrime) at Bundesanstalt für Materialforschung und–prüfung (BAM) in Berlin. An aliquot of homogenised untreated and treated guano and homogenised fishmeal samples were weighed into tin capsules and transferred to the auto-sampler. According to the 2014 IUPAC technical report [66] the δ^13^C and δ^15^N values are reported relative to Vienna Pee Dee Belemnite (VPDB) and AIR, respectively. The reproducibility of analyses was controlled with an in-house casein standard (Merck KGaA) that was repeatedly analysed (n = 30) at the start and within each session. The arithmetic mean of the average values from 4 sessions (days) and the standard deviation were δ^15^N 6.34 ± 0.08 ‰ (1SD), δ^13^C -21.86 ± 0.02 ‰ (1SD), %N contents 14.6 ± 0.1% (1SD), and %C contents 49.0 ± 0.3% (1SD), respectively.

Concentrations of non-volatile elements of selected treated guano and fishmeal samples (S1 Table and S2 Table) were analysed by ICP-OES (inductively coupled plasma optical emission spectrometry) with a Varian Vista Pro instrument in the laboratory of the Sediment Geochemistry Group at MARUM, University of Bremen. All guano and fishmeal samples were prepared by dissolving 50 mg of homogenised aliquots in 3 ml of double distilled HNO_3_. Certified high purity multi element standards were used for element-specific instrumental calibration. Three replicates were analysed and the relative analytical uncertainties were better than 4% (2σ) for all element concentration determinations except for aluminium (approximately 20%) and barium (approximately 10%).

The statistical evaluation of correlations between isotope ratios and elemental compositions (guano) or spatial distribution (fishmeal) is given at the 95% confidence level, and we report the significance (p) and number of samples (n) in the text.

## Results

### ^14^C systematics of guano and fishmeal and the age of the guano

Guano from selected sample sites was dated by ^14^C at the Poznan Radiocarbon Laboratory by accelerator mass spectrometry (Table 1). A major issue in dating marine materials by ^14^C methods is the correction of analysed data for carbon-additions from a marine reservoir, especially in upwelling regimes [67]. Two samples from the lower section of profile Mej.1 indicate a post-bomb signature [68] of 103.95 pMC at 43 mm depth and 104.32 pMC at 50 mm depth, respectively (pMC, percent Modern ^14^C, means all data exceeding 100% threshold of natural ^14^C abundance show the effect of the bomb signal that built up artificially generated ^14^C in the atmosphere, starting with the atmospheric nuclear weapon test in the 1950^s^, peaking in the mid-60^s^, and ceasing after the implementation of the atmospheric test ban from 1963 [68, 69]). The stratigraphically older sample is slightly more radiogenic, i.e. the formation of the Mej.1 samples is at the young side of the decaying atmospheric post-bomb signal near the 2011 AD end of the bomb signal’s activity curve [68]. We assume young ages around 5 years that also appear to be reasonable from the depositional environment below an active nesting site near sea level. Location Mej.3 represents a resting place where two samples from 15 mm depth show post-bomb signatures of 104.82 pMC and 105.29 pMC, respectively. These values are slightly older than those at Mej.1, if we assume a position of the Mej.3 samples at the end of the activity curve and not at the build-up branch of the signal in the 1950^s^ [68]. The two options cannot be distinguished in Mej.3 without samples from different depths in the same profile. We also analysed ^14^C in fishmeal, which represents the feeding level of the seabirds. The three fishmeal samples of catches from 2014 AD are the youngest analysed material and have accordingly 102.23, 102.32, and 100.99 pMC. The modern guano at two locations and the fishmeal indicate modern atmospheric ^14^C values, without prominent contributions from an older marine reservoir. The %C content of the guano samples derives from the prey of the birds, i.e. the fish. If the ^14^C compositions of the fishmeal, Mej.1, and Mej.3 locations are not transitional, we have also to consider a possible atmospheric ^14^C signature in the older guano samples. Therefore, we decided to present calibrations to the marine [70] and the atmospheric ^14^C evolution of the southern hemisphere using SHCal13 [71]. The samples were calibrated assuming a 100% marine or a 100% atmospheric ^14^C signal using OxCal version 4.2 software [72]. Correction to the regional seawater reservoir is not unequivocal. A uniform reservoir age ΔR of 190 years was assumed in earlier work for the Pacific coast of South America [73], but reservoir ages in the upwelling zone off Chile and Peru appear to be rather variable along the time axis due to changing contributions from deep seated ‘old’ sources [74, 75]. We use the weighted average of six ΔR values of 200 ± 140 years between 20 and 23° S for regional marine reservoir correction [74].

The assignment of locations to modern guano is founded on the observation of ongoing deposition and ^14^C systematics. The profile Mej.1 is not older than approximately 5 years, whereas the deposit at the resting place Mej.3 is slightly older: either early post-bomb or late post-bomb. The profile at resting place Mej.2 indicates increasing ^14^C calendar ages with depth of 1860 ± 70 AD at a 7.5 mm depth using the marine reservoir correction or 1550 ± 50 AD calibrating to the SHCal13 atmospheric curve [67]; respective values at a 36 mm depth are 1490 ± 130 AD and 1040 ± 55 AD, and at a 55 mm depth 1335 ± 125 AD and 850 ± 65 AD.

The ages of fossil guano are given as maximum ages referring to the ages of the respective terraces in the geological description above. The ^14^C dating at the Punta Negra resting place reveals a much younger age than the maximum age of 100 ka from the terrace. Two samples have calibrated ^14^C calendar dates of 1460 ± 125 AD near the surface at a 20 mm depth and 565 ± 180 BC at a 320 mm depth near the bottom of the profile using the marine reservoir correction or 990 ± 30 AD and 295 ± 75 BC using SHCal13 [71], i.e. the age increases with profile depth.

### Isotopic composition of modern guano

**Fig 3:**
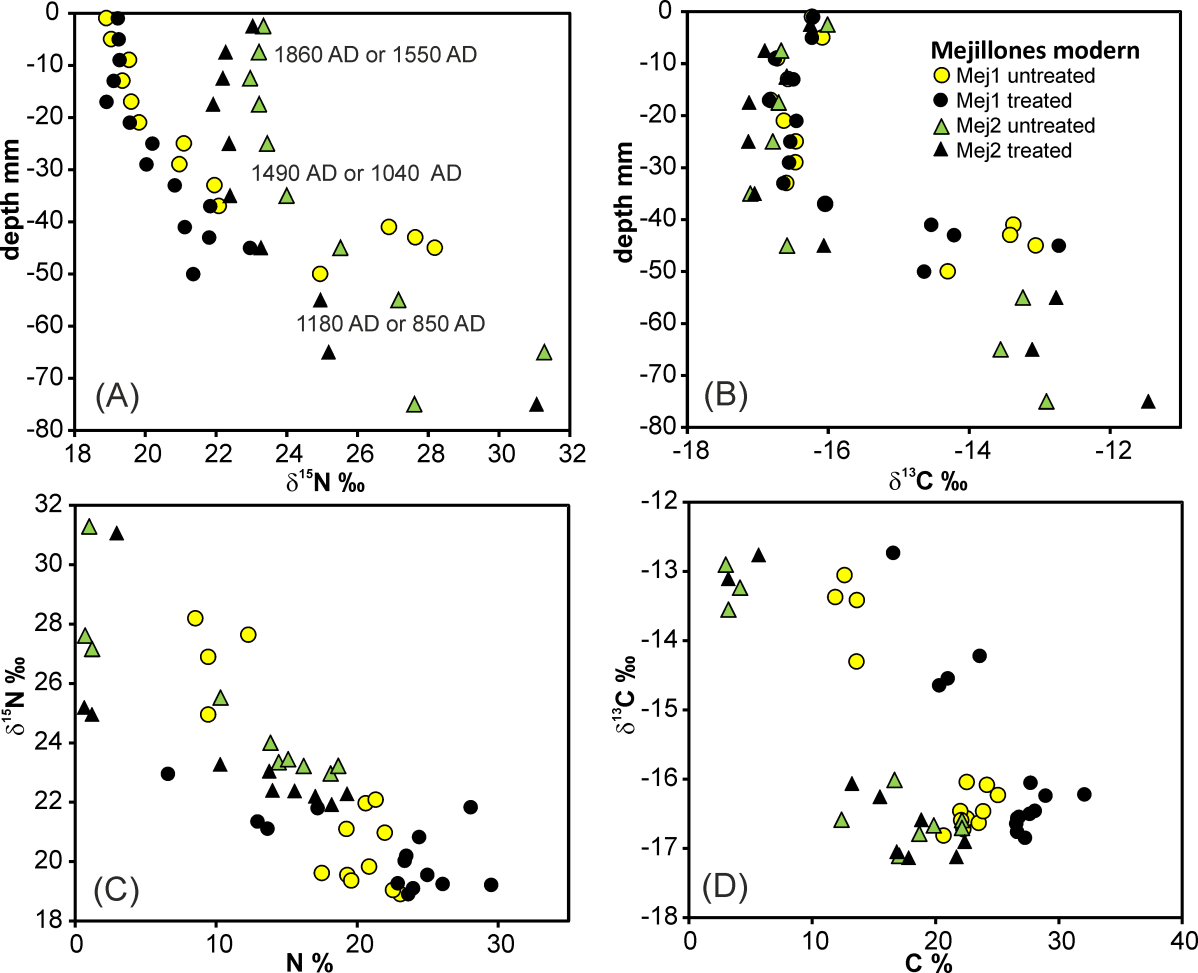
Isotopic and elemental composition of modern guano at Mej.1 and Mej.2. (A, B) Relations between δ^15^N and δ^13^C and depths of profiles. (C, D) Relations between δ^15^N and δ^13^C and %N and %C elemental contents.

The δ^15^N values of treated and untreated guano from high resolution sampling at Mej.1 show a range from 19 to 28‰. The values are either constant or slightly decreasing along the first 20 mm profile before starting to evolve towards more positive values that increase with age, meaning depth (Fig 3A; S1 Table). The shift of δ^15^N with depth in untreated guano by up to 9‰ is larger than in treated samples, which show a shift up to 4‰. The uppermost untreated and treated samples are similar in their δ^15^N. Location Mej.2 starts with a heavier δ^15^N value (23‰ instead of 19‰ at Mej.1) and evolves with increasing depth at a shallower gradient to values up to 31‰. All but one of the treated samples are isotopically lighter than the untreated ones (Fig 3A). It should be mentioned that the sedimentation rate at the nesting site Mej.1 is much higher than at the resting site Mej.2 according to the ^14^C ages (Fig 3A).

The δ^13^C values in the Mej.1 and Mej.2 profiles vary between -17‰ and -13‰. They form roughly two clusters between -17‰ and -16‰ down to approximately 40 mm and between -14.5‰ and -12.5‰ in the lowermost sections (Fig 3B). In contrast to δ^15^N, the δ^13^C becomes slightly but systematically lighter down to 40 mm depth before evolving to heavier δ^13^C below 40 mm depth in both profiles. The differences in δ^13^C between the profiles and between treated and untreated samples are small.

**Fig 4:**
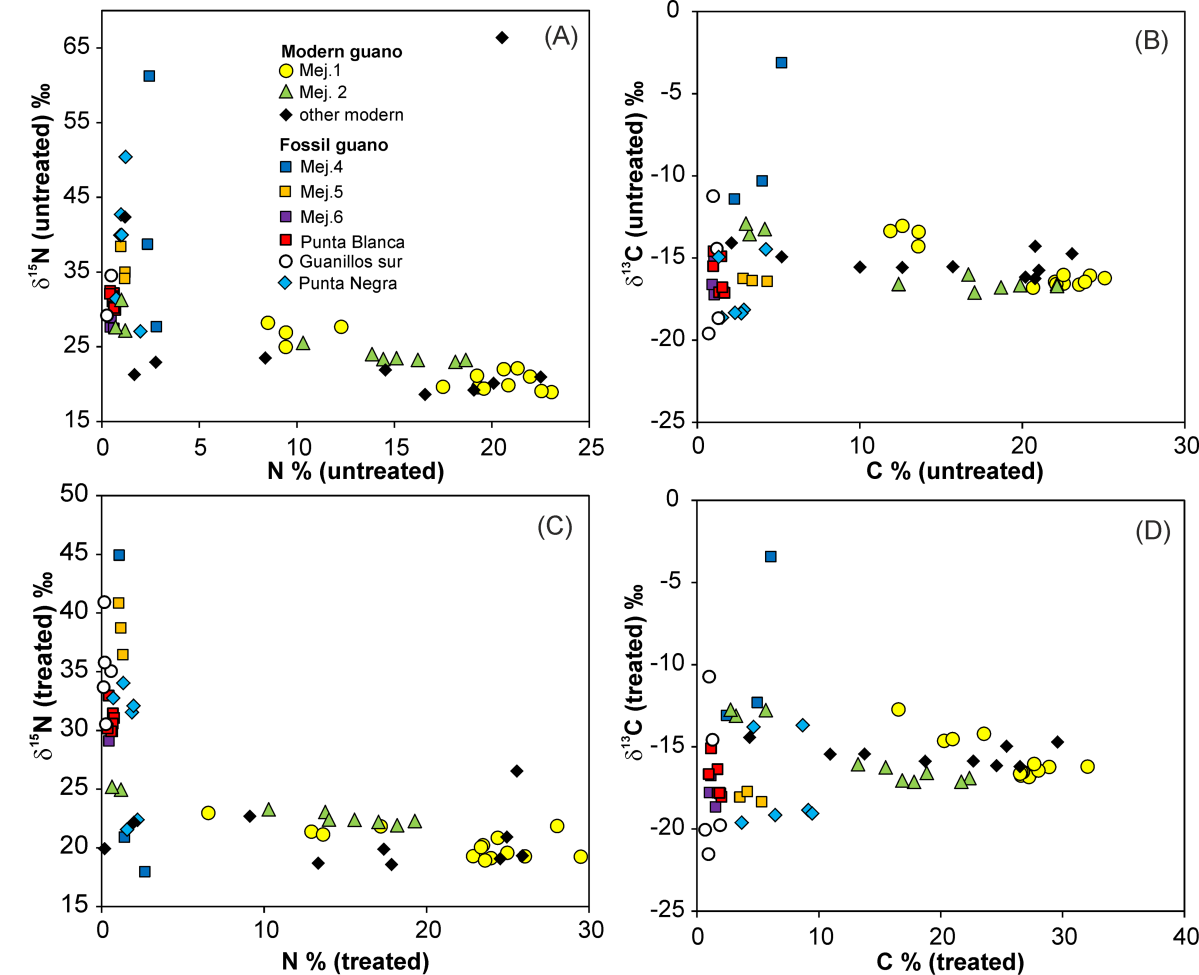
Isotopic and elemental composition of fossil and modern guano. (A-D) δ^15^N and δ^13^C versus %N and %C contents of (A, B) untreated and (C, D) treated samples.

The values of δ^15^N linearly increase with decreasing contents of %N (Figs 3C and 4) in profile Mej.1 (untreated: p = 2.3^−5^, n = 14; treated p = 6.0^−3^, n = 14) and Mej.2 (untreated: p = 7.7^−4^, n = 9; treated p = 2.0^−2^, n = 9). Also, δ^13^C increases with decreasing contents of %C (Figs 3D and 4; Mej.1 untreated: p = 1.3^−6^, n = 14; treated p = 7.9^−5^, n = 14; Mej.2 untreated: p = 4.3^−4^, n = 9; treated p = 1.1^−4^, n = 9).

The reproducibility (1SD) of δ^15^N and δ^13^C analyses is better than 0.1‰. For %C and %N analyses it is better than 0.4%, and hence smaller than the size of the symbols in the Figures.

The group of “other modern guano” samples is collected from the surface at various locations (S1 Table; Mej.1a, Mej.3, and south of Antofagasta). The δ^15^N and δ^13^C compositional ranges of these samples are similar to the ranges of Mej.1 and Mej.2 samples (Fig 4; S1 Table). Two untreated samples display very heavy δ^15^N of 43‰ and 66‰ (Mej.1a), but the related treated samples show common values of 26‰ and 20‰, respectively. Linear correlations between %N contents and δ^15^N (untreated p = 0.5, n = 11; treated p = 1, n = 11) and %C contents and δ^13^C (untreated/treated p approximately 0.2, n = 11) are absent in the “other modern guano” group (Fig 4).

### Isotopic composition of fossil guano

The δ^15^N values for fossil guano at Mej.4, Mej.5, Mej.6, and locations north of the Mejillones Peninsula scatter between 27‰ and 62‰. The lightest δ^15^N in untreated samples of fossil guano resemble the heaviest δ^15^N found in the untreated modern guano. Only four treated fossil guano samples have comparably light δ^15^N values within the compositional range of modern treated samples (Fig 4). Nitrogen contents are below 3 wt%, and in most samples close to or below 1 wt% (Fig 4; S1 Table). The δ^15^N values and elemental contents are not correlated with depth at *in-situ* profiles Punta Blanca and Guanillos, but δ^13^C shows variation to somewhat heavier compositions at depths shallower than 30 cm. At the Mejillones Peninsula, the resedimented deposits Mej.5 and Mej.6 on old Mejillones terraces show small compositional ranges, but the largest variations of δ^15^N and δ^13^C occur in the *in-situ* deposit Mej.4 (Fig 4).

The δ^13^C in untreated and treated fossil guano (except for the heavier Mej.4) is similar or lighter than in modern guano (Fig 4B and 4D) at %C contents <10 wt% and 5 wt%, respectively.

### The influence of leaching on the C and N isotopic and elemental composition

**Fig 5:**
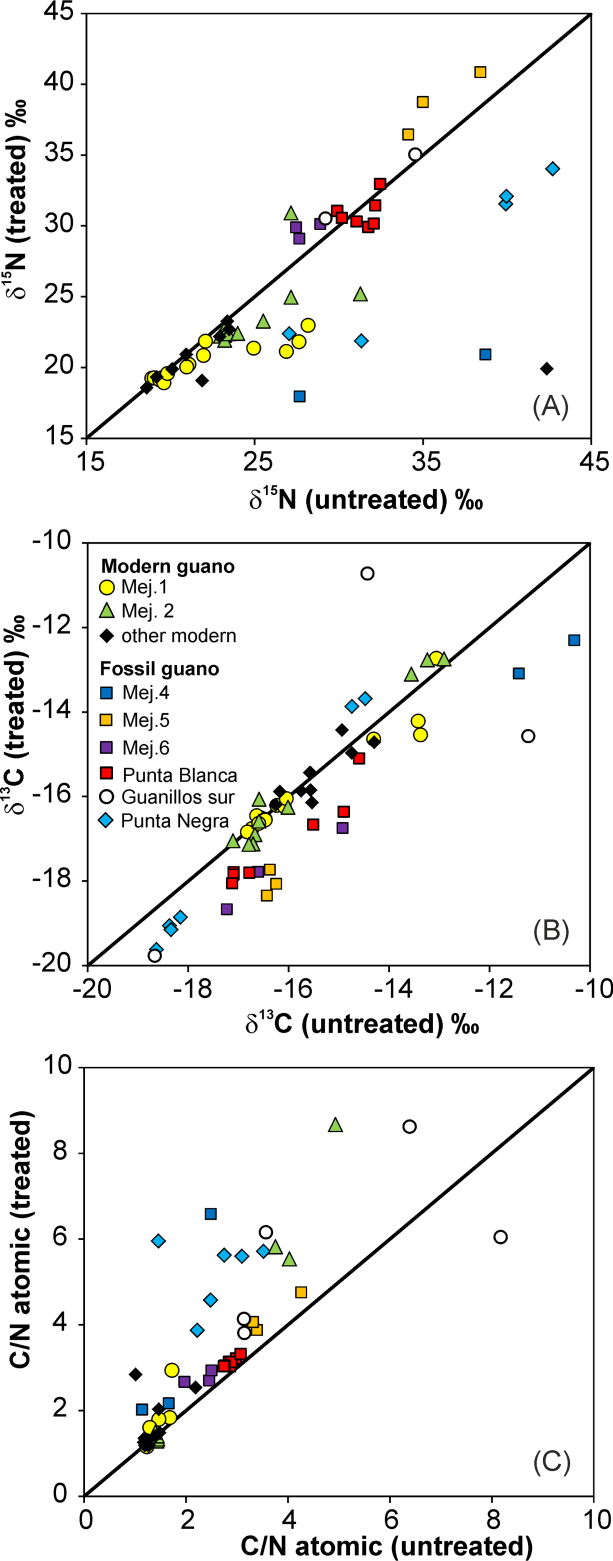
Modern and fossil guano: Influence of the leaching procedure. Comparison between (A) δ^15^N, (B) δ^13^C, and (C) C/N in treated and untreated samples.

The large differences in δ^15^N between some treated and untreated sample pairs of modern guano point to the presence of isotopically heavy, water-soluble N-compounds in these untreated samples (Fig 5A). In fossil guano, differences in the δ^15^N between treated and untreated samples are small: within ±1‰ along the 1:1 distribution line with only a few exceptions (Fig 5A). The variation of δ^13^C between treated and untreated sample pairs is minimal in modern guano. In fossil guano, it deviates by approximately 1 to 2‰ from the 1:1 distribution line towards heavier compositions in many untreated samples (Fig 5B).

The higher %N and %C contents in many treated samples, especially of the modern guano compared with their untreated counterparts, is caused by removal of the water soluble fraction, mainly NaCl during the leaching procedure (S1 Table). The molar C/N ratios of many treated/untreated pairs are close to 1:1 (Fig 5C). The deviation of C/N from the 1:1 line mainly indicates a loss of N in modern and fossil guano during the leaching procedure. This effect is strong in some of the modern guano samples (Fig 5C).

The molar C/N ratios in many treated and untreated samples of modern guano are close to approx. 1.2, which is the composition of uric acid. The value evolves in modern guano to values not exceeding 1.5 in most samples (S1 Fig). This directly indicates that urea (C/N = 0.5) does not play a major role in the composition of the guano.

### Elemental composition of modern and fossil guano

**Fig 6:**
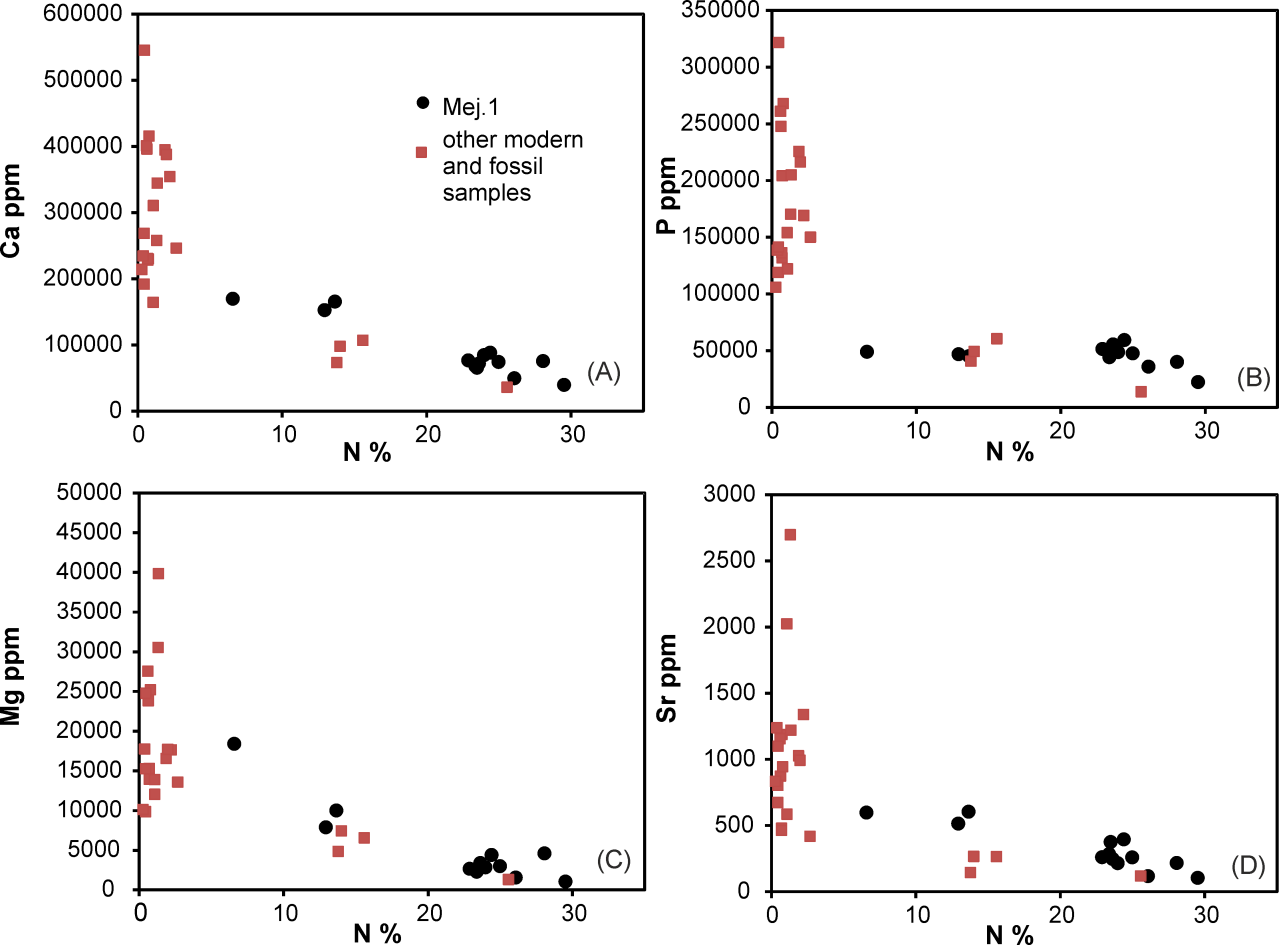
Contents of non-volatile elements versus %N contents. Non-volatile (A, B) major-, (C) minor-, and (D) trace-element contents increase with decreasing %N (and %C, not shown) contents.

In the high resolution profile Mej.1, non-volatile elements account for less than 10 wt% in the youngest and freshest uppermost sampled layers, and increase with depth, i.e. age to up to 25 wt% (S1 Table). The non-volatile element budget in treated modern guano is dominated by Ca and P, and both elements typically account for more than 80% of the non-volatile elements. The increase of non-volatile-element contents correlates with decreasing %N (Fig 6) and %C (not shown) contents in the Mej.1 profile, a trend which is followed by other modern and fossil guano samples (Fig 6A–6D: S1 Table).

Treated fossil guano has %N contents <3 wt%, most samples <2 wt% (Figs 4C and 6). The non-volatile elements in fossil guano account for typically more than 40 wt% in the treated samples due to the massive loss of C and N compounds. This passive enrichment process leads to high P concentrations between 10 and 32 wt% (Fig 6A–6D).

### Isotopic and elemental composition of fishmeal

**Fig 7:**
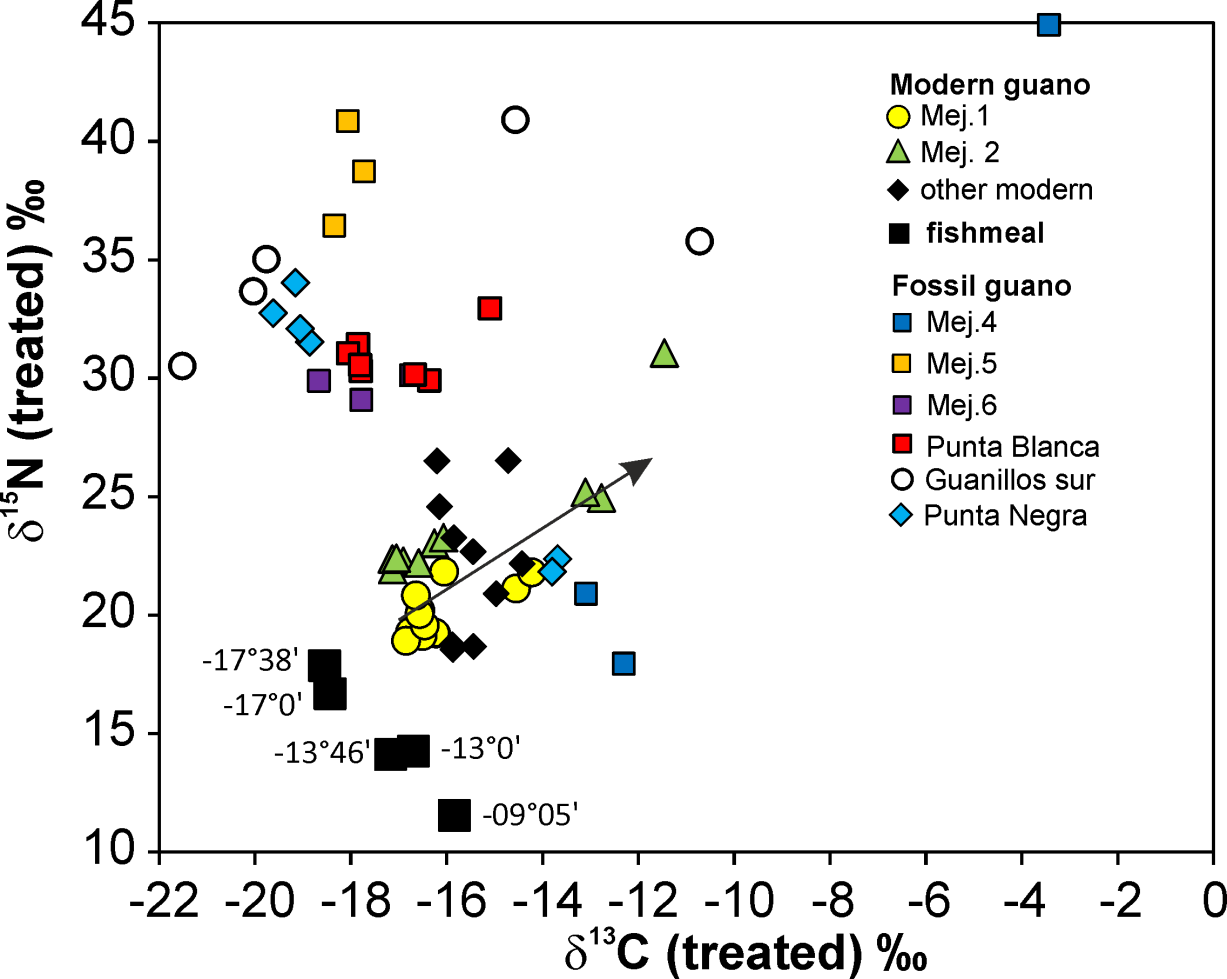
δ^15^N and δ^13^C composition of fishmeal (anchovy) and guano. Fishmeal: Latitudes of factories and a single sample location of anchovy data at 13°46’ from [76]. Modern and fossil guano: Correlation between δ^15^N and δ^13^C is restricted to the in-situ profiles Mej.1 (p = 2.9^−4^; n = 14) and Mej.2. (p = 1.1^−3^; n = 9), and Punta Negra (p = 1.8^−4^; n = 6). The arrow indicates increasing depth in profiles Mej.1 and Mej.2.

The fishmeal samples show a systematic latitudinal increase in δ^15^N from +11.4 to +17.9‰, and a latitudinal decrease in δ^13^C from -15.8 to –18.5‰ between 9 and 17° S (Fig 7, S2 Table; for δ^13^C - latitude see S2 Fig; p = 2.9^−12^, n = 18; δ^15^N-latitude p = 1.7^−11^, n = 18). Accordingly, δ^15^N and δ^13^C in the fishmeal are significantly correlated (p = 6^−14^; n = 18).

The major element composition including C, N, Ca, P, Na and some trace elements (Sr, Mn, S, Si, K) of selected samples is rather uniform (S2 Table). The molar C/N ratio is 4.38 ±0.07 (1 SD; n = 18; S2 Table) and far removed from the ratios of 1.2 to 1.5 in many modern guano samples.

## Discussion

### Baseline N and C isotopic composition of modern guano

**Fig 8:**
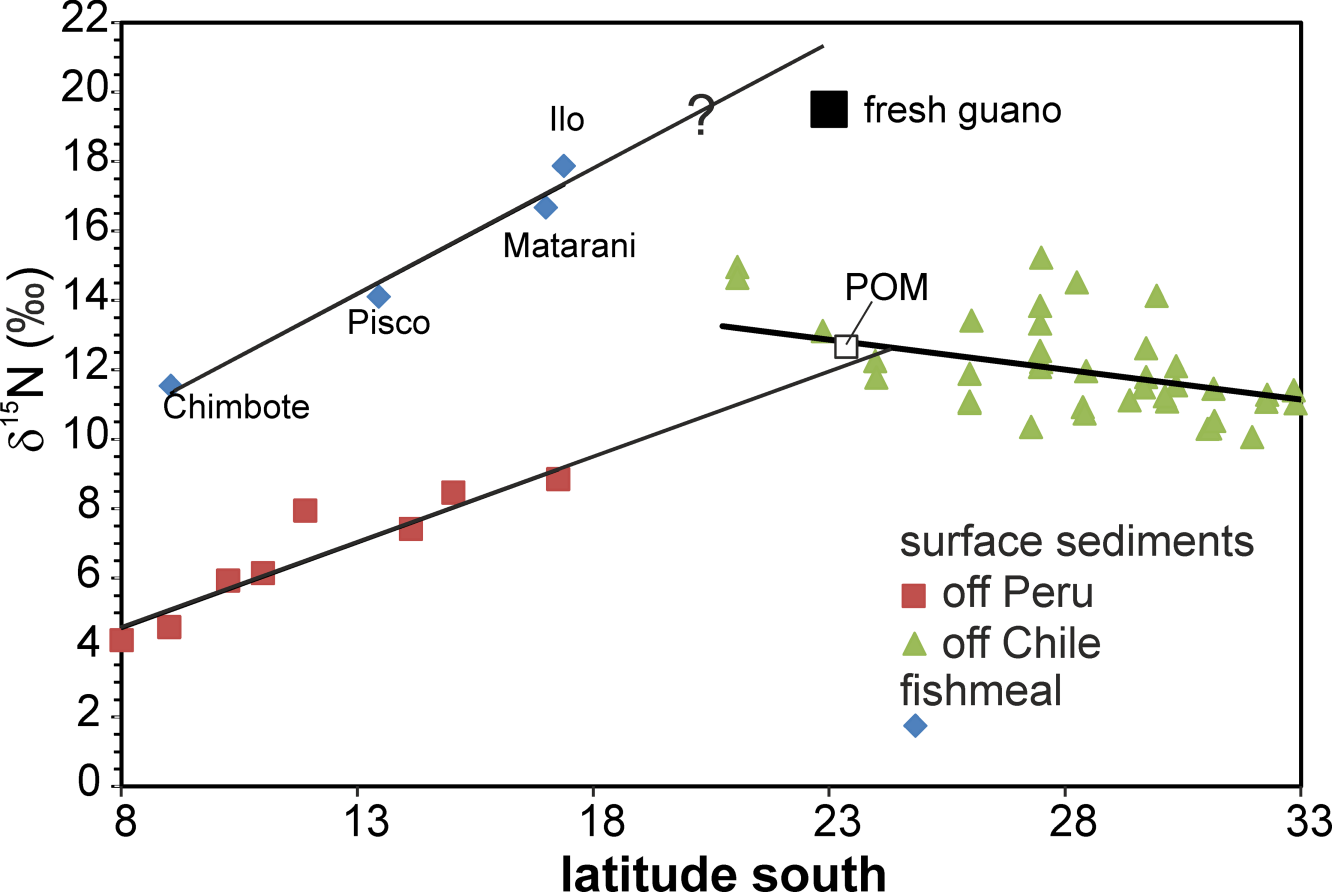
Latitudinal variation of N isotopic composition in fishmeal and marine surface sediments. The average fishmeal composition (*Engraulis ringens*) is labelled with the respective production site. The isotopically lightest modern guano occurs at the Mejillones Peninsula. Modern particulate organic matter (POM) is off Mejillones at 23° S [79]. The composition of surface sediments from the shelf between 8° and 18° S are average values at the respective latitude [43], whereas single analyses are shown between 22° and 33° [41, 42].

The N and C isotopic signature of modern guano should be directly linked to the composition of the bird’s preferred prey, the Peruvian anchovy (*Engraulis ringens*), i.e. should reflect the feeding level of the birds. The enrichment of ^15^N in the marine trophic chain is about 3±1‰ increase of δ^15^N between food and consumer [3, 15, 77]. The fishmeal (anchovy) shows a strong systematic latitudinal increase of δ^15^N towards the South (δ^15^N from +11.4‰ to +17.9‰), which follows the increase of δ^15^N in surface sediments from the shelf along the same latitudes off Peru. The distances of fitted lines through fishmeal and shelf surface sediment δ^15^N data are approximately +6.2‰ at 9° S and +8.0‰ at 17° S (Fig 8; [43]). The only published composition of anchovy indicates δ^15^N of 14.2‰ and δ^13^C of -16.7‰ (13°46’ S; [76]), and coincides with our fishmeal data near this latitude (Fig 7). The difference between fishmeal δ^15^N and the δ^15^N of surface sediments off Peru is close to two marine trophic levels (each with δ^15^N +3±1‰). This observation is in accordance with the fact that (1) the anchovy feeds largely on zooplankton [39, 40], which is at least one trophic level above phytoplankton and (2) that phytoplankton constitutes the bulk of particulate organic matter (POM) that sinks to the seafloor and becomes incorporated into the surface sediments [12]. The relatively uniform difference of δ^15^N between fishmeal and surface sediments indicates that latitudinal isotopic variations already occur in the phytoplankton and are passed through the food web to the non-migratory fish. Time slices represented by the composition of the fish are likely much shorter than those represented by the shelf surface sediments, and could reflect short lived changes of δ^15^N in the upwelling system. Such transitional change could be reflected by the increasing difference in average δ^15^N towards the South between fishmeal and sediment (Fig 8).

Fishmeal δ^15^N and δ^13^C data are not available in the Chilean section, but surface sediments of the shelf indicate heavier δ^15^N towards the North along the Chilean coast up to 21° S [41, 42]. The regression lines through the Chilean (p = 2.2^−23^; n = 105) and Peruvian (p = 3.2^−22^, n = 106) shelf-surface sediments indicate δ^15^N of approximately 13.5‰ at 21° S and approximately 9‰ at 17° S. Therefore, we assume that the heaviest δ^15^N occurs somewhere between 18 and 21° S, where no sediment data exist (Fig 8). The δ^15^N of the birds’ excreta should be isotopically lighter than the feed, or at maximum close to the feed, depending on the fractionation and mass balance of N between the proportion that is incorporated at the higher trophic level of the birds and the excreted proportion [15]. The comparison of the C/N ratios in fish tissue and guano precludes a possible major contribution of undigested fish to the modern guano samples. Fish tissue has variable C/N but generally > 3 [78], and the analysed fishmeal has an average molar C/N of 4.38±0.07 (1SD; n = 18; S2 Table). Most modern guano samples show molar C/N ratios between 1.2 and 1.5, which are close to the molar C/N ratio in uric acid of 1.22. This indicates the dominance of uric acid element ratios, which comprises 50–80% of fresh guano [61–63]. Similar C/N values in fresh guano and separates of uric acid from the same guano are reported for North Atlantic seabirds [15]. Therefore, we consider the difference of 6‰ or 2 trophic levels between the isotopically lightest modern guano at 23° S with δ^15^N of 19‰ and the surface sediment from the shelf with 13‰ as a minimum value (Fig 8). The latter value is also matched by modern POM from the same latitude (Fig 8; [79]). The inferred difference of 6‰ is similar to the difference between fishmeal and surface sediments in the Peruvian section. Only the samples from the uppermost layers of Mej.1 (treated and untreated samples Fl-203—Fl-211; S1 Table, Fig 3A) and two samples out of the collection of nine “other modern guano” samples from the surface of observationally active resting places plot close to potentially pristine δ^15^N values (Figs 7 and 8). All other samples of modern guano are already affected by severe changes of δ^15^N.

The baseline for the C isotopic composition of the birds’ food faces the same problem as for N isotopes. The fishmeal shows a systematic latitudinal variation in C isotopes with lighter δ^13^C towards the South (S2 Table; S2 Fig). However this trend is not seen in the δ^13^C of Peruvian shelf-surface sediments, which scatter independent of the latitude between -20‰ to -21‰ (S2 Fig; [80]). We speculate that the systematic change in δ^13^C,unlike δ^15^N, does not occur in the phytoplankton but higher in the food chain.

The ^13^C enrichment in marine trophic chains is much smaller than the increase of ^15^N, and therefore easily masked by the compositional variability of food sources [81, 77]. The δ^13^C variability of many modern guano samples (treated and untreated) is within the variation of the fishmeal δ^13^C data (Fig 7).

### Guano diagenesis: Decay of N-compounds and ^15^N enrichment

The initial %C and %N content of modern guano is defined by the composition of the birds’ dominant metabolic waste, the uric acid and potential contributions from undigested prey. Therefore, the pristine guano compositions should be similar at all locations considering the birds’ specialized alimentation. The systematic change in element concentrations and N and C isotopic composition with increasing depth (age) at profile Mej.1 indicates that the compositional variation of modern guano must be related to post-depositional changes.

Hydrolytic decomposition of various N-compounds in organic materials including uric acid is widely accepted as being at least partly a result of microbial activities [82, 83]. This starts immediately after deposition and is indicated by the strong ammonia smell from NH_3_ release in sea bird rookeries (own observation; [17, 19]), and cattle and poultry manure deposits [84, 85]. The decomposition of uric acid is quick under aerobic and slower under anaerobic conditions [62, 86]. Both processes require the presence of water [82, 87]. The present climate in our study area is nearly void of any rainfall and the average annual precipitation between 1970 and 2000 at the Pacific Coast was 3.4 mm in Antofagasta and even lower towards Iquique in the North (Fig 1A; [88]). This generally dry climate is considered to be long-lasting, having started in the late Miocene [26–30]. While water for material transport from or within modern guano deposits can be excluded, moisture supply to the surface is provided by sea spray and occasional drizzle from seasonal fog at the coast and by the addition of fresh guano. To our knowledge, the viability of the microbial decomposition of N-compounds has not been studied for such dry conditions. However, microbial activity could be confirmed even in soils of the extremely dry desert inland from the Pacific coast of Northern Chile [89, 90]. Plant-remains in ornithogenic soils that can influence the δ^15^N systematics in more humid climates [22, 91–93] are absent in our samples. Regardless of the actual process or driver of the decomposition of N-compounds, the δ^15^N values increase with age and decreasing %N contents in modern guano profiles, and evolve to very heavy δ^15^N at low %N contents in fossil guano.

The fractionation of N isotopes between residuum–dissolved NH_3_ –degassing NH_3_ has been studied in various natural and synthetic materials. Strong isotope fractionation between NH_4_ and NH_3_ in synthetic aqueous solutions enriches the ^15^N in the residual NH_4_ in an experimental study. Here, the progressive NH_4_ decomposition is related to increased pH (>>6) in the solute [94]. Similar trends towards the enrichment of ^15^N in residual N-phases were observed in natural systems. Studies on the compositional evolution during the decomposition of the primary N-compounds of seabird guano [18, 19], and chicken and cattle manure [62, 95, 96] indicate the volatilisation of N as NH_3_ (ammonia gas) and fixation as ammonium (NH_4_) salts, minor nitrite (NO_2_) and nitrate (NO_3_) salts, which starts immediately after deposition. Generally, during the decomposition of N bearing organic compounds, nitrogen is easily volatized as NH_3_ gas and ^14^N preferentially partitions into the gas phase, increasing the δ^15^N of N bearing residual solids [19, 18, 62, 92]. A direct proof for such processes in our modern guano samples is given by the isotopic composition of treated and untreated samples. The upper-most layers of Mej.1 show nearly identical δ^15^N in treated and untreated samples. The difference between treated and untreated samples becomes larger with increasing depth towards heavier δ^15^N in the untreated samples, i.e. ^15^N is partitioned into water soluble N bearing salts that are preserved in the extremely dry climate and removed by the water treatment in the laboratory. This effect becomes more important with increasing age (depth) in the profiles Mej.1 and Mej.2, and is also strong in samples with a comparably short geological history, as in the group “other modern guano”. The increase of δ^15^N relates to decreasing %N contents, and is roughly conform to a Rayleigh type process [97] in the profiles Mej.1 and Mej.2 (S3 Fig).

The location Punta Negra was classified as fossil in the field, but it has high contents of water soluble fraction throughout the depth profile. The ^14^C ages at 30 mm depth at Mej.2 and at 20 mm at Punta Negra are similar (Table 1), and the δ^15^N values coincide at these depths (S1 Table). The δ^15^N in the profile Punta Negra starts off comparably light in the two close-to-surface samples and becomes heavier with increasing depth (S4 Fig). The trends are similar in treated and untreated samples of the Punta Negra profile, despite treated samples being approximately 5 to 18‰ lighter.

Fossil guano from various locations (except for Mej.4) shows heavy δ^15^N at low %N contents. The heaviest δ^15^N in modern guano profiles coincides with the lightest δ^15^N of fossil guano (Figs 4–6). The heavy δ^15^N generally fits the observation from the modern *in-situ* profiles that the decomposition of N-compounds by NH_3_ removal is a function of age and results in heavier δ^15^N. The differences between treated and untreated samples are small in most fossil samples, but in contrast to the modern samples some fossil samples show slightly heavier δ^15^N in the treated samples (Fig 5A). This may be attributed to the redistribution of mobile N-salts with heavy δ^15^N by aqueous fluids during the long history of the fossil deposits. Material transport by water is also inferred from the removal of halite at most fossil guano locations, which is indicated by the low contents of water soluble material (S1 Table). The fossil guano has obviously gone through more humid periods with pluvial water available that removed/reduced the water soluble fraction in the sediment column.

### Guano diagenesis: δ^13^C systematics

Strong variations of δ^13^C in modern guano between -26‰ to -28‰ are restricted to deep sections in profiles Mej.1 and Mej.2, i.e. they are related to the ageing of the guano (Fig 3B). The increase of δ^13^C is correlated with decreasing %C contents in the depth-profiles and in samples of the “other modern guano” group (Fig 5B and 5D). This suggests the extraction of C as CO_2_ during decomposition. The δ^13^C of fossil guano is comparable or slightly lighter than in modern guano, and unlike the δ^15^N evolution, it does not represent the continuation of the δ^13^C trend in profiles Mej.1 and Mej.2. The differences between treated and untreated samples are generally small. The evolution of δ^13^C with increasing depth in the Punta Blanca and Punta Negra profiles starts with heavy compositions near the surface and continues to lighter δ^13^C at the deeper points (S4 Fig). This is in contrast to the observations in Mej.1 and Mej.2, and we speculate about an exchange with heavy atmospheric CO_2_ in the upper part of the Punta Blanca and Punta Negra profiles. The δ^13^C of treated samples in fossil and modern guano are with few exceptions slightly lighter than in untreated samples. In contrast to modern guano, where uricite is the main C-bearing phase, oxalates (e.g. whewellite) are the principal C-bearing minerals in fossil guano. The distribution of C isotopes during the formation of whewellite was described for various occurrences (but not for fossil seabird guano) and appears to be variable with a large range of δ^13^C compositions and a trend to lighter δ^13^C in organic sources [98].

### The fate of released N

The guano represents a constant flow of material with high %N contents from sea to continent. The age and composition of the deposits in Northern Chile from the Pliocene onwards indicate a longstanding process of N emissions as a result of guano degradation. Nitrogen is mainly lost to the atmosphere in the form of gaseous N-components, and might at least impact the NH_3_ supply near and downwind from guano deposits [99]. The impact on plant ecosystems via air transport of reactive NH_3_ in the subarctic climate has been demonstrated on a short range (some km, [100]). Speculations dating back to the 19^th^ century on the formation of the once economically important high grade nitrate deposits mainly east of the North Chilean Coastal Cordillera (Fig 1A) include the atmospheric deposition of N from guano degradation at least as a contribution to the nitrogen enrichments in soils (for a review see [101]). This process cannot be excluded from more recent research on the nitrate [102]. The impact of longstanding NH_3_-release in the generally (hyper)arid climate of Northern Chile still awaits investigation.

## Conclusions

Seabird guano has been preserved from at least the Pliocene onwards at the Pacific Coast of Northern Chile and Southern to Central Peru, and represents a potential archive for the N and C isotopic signatures of marine organic matter/sources through time. Our study reveals that N and C isotopic and elemental compositions are subject to fast secular alteration, which occurs within years even in the fully arid climate of Northern Chile. Despite a general evolution towards heavier δ^15^N at decreasing %N contents, the systematics of this alteration (diagenesis) is not uniform along the time axis (age) of the deposit. This precludes any inferences from (sub)fossil guano on δ^15^N and δ^13^C variations in past marine food chains. Unaltered, fresh guano could provide a useful tool for the study of marine signatures [15]. However, diagenesis (alteration) is not easily detected and no threshold exists for freshness except immediate collection after deposition. Furthermore, guano-birds restrict their marine alimentation exclusively to non-migratory schooling fish, but the δ^15^N and δ^13^C composition of the fish and hence its food chain is presently highly variable. This has to be considered even for a contiguous system like the present Pacific coastal waters (Fig 8).

On the other hand, the δ^15^N in guano quickly evolves to very heavy compositions that are rarely found in other earth materials [2]. This makes guano fertilization a good tracer in actual and fossil natural ecosystems [21] or man-made agricultural ecosystems [33–35] for the impact of marine material. The signal from crops is potentially passed on to their consumers as shown in archaeological studies [34]. Our survey of fossil guano in Northern Chile reveals very heavy δ^15^N (>27‰ up to 60‰).

## Supporting information

S1 Table Results of C and N isotope and elemental analyses of untreated and treated guano samples and elemental analyses of selected samples by ICP-OES. (XLS)

S2 Table Results of C and N isotope and elemental analyses of fishmeal. (XLS)

S1 Fig Molar C/N versus (A) δ^15^N and (B) δ^13^C shows the proximity to uric acid composition. Five very high C/N ratios (S1 Table) are not shown.
(TIF)

S2 Fig The δ^13^C composition of fishmeal (anchovy) is strongly correlated with the latitude. Surface sediments of the same latitudinal section show a small scatter but no correlation with latitude [80].
(TIF)

S3 Fig Rayleigh type plots of δ^15^N versus ln (N wt%) are poorly correlated in (A) profile Mej.1 and (B) profile Mej.2.
(TIF)

S4 Fig (A-C) Fossil guano: the correlation of δ^13^C and δ^15^N of treated and untreated samples with increasing depth. (A) In Punta Blanca the δ^13^C decreases with increasing depth whereas δ^15^N (not shown) is not correlated. In Punta Negra (B) δ^15^N is lighter and (C) δ^13^C is heavier in the uppermost layer, but both isotope ratios show little variation between -6 cm to –32 cm. (B, C) The two close-to-surface samples are taken in a horizontal distance of 1 m at the same depth; especially the treated samples show similar comparably light δ^15^N and heavy δ^13^C.
(TIF)
