# Supplementary figures and images for: The stable isotope composition of nitrogen and carbon and elemental contents in modern and fossil seabird guano from Northern Chile – Marine sources and diagenetic effects
Source: PLoS One. 2017 Jun 8;12(6):e0179440. doi: 10.1371/journal.pone.0179440 (PMC5464657; doi:10.1371/journal.pone.0179440)

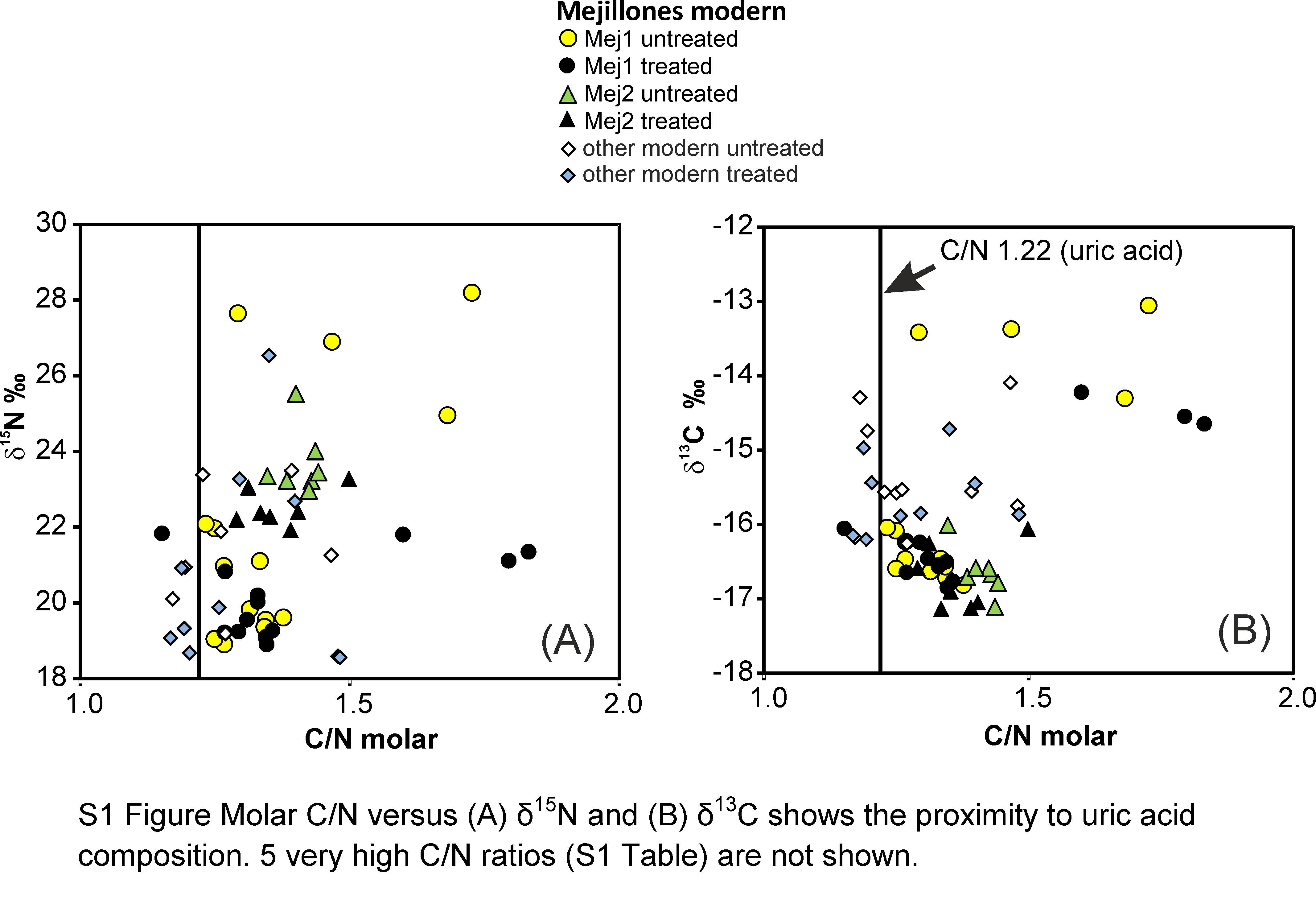

Supplement: S1 Fig — Molar C/N versus (A) δ15N and (B) δ13C shows the proximity to uric acid composition. Five very high C/N ratios (S1 Table) are not shown. (TIF) [file pone.0179440.s003.tif]

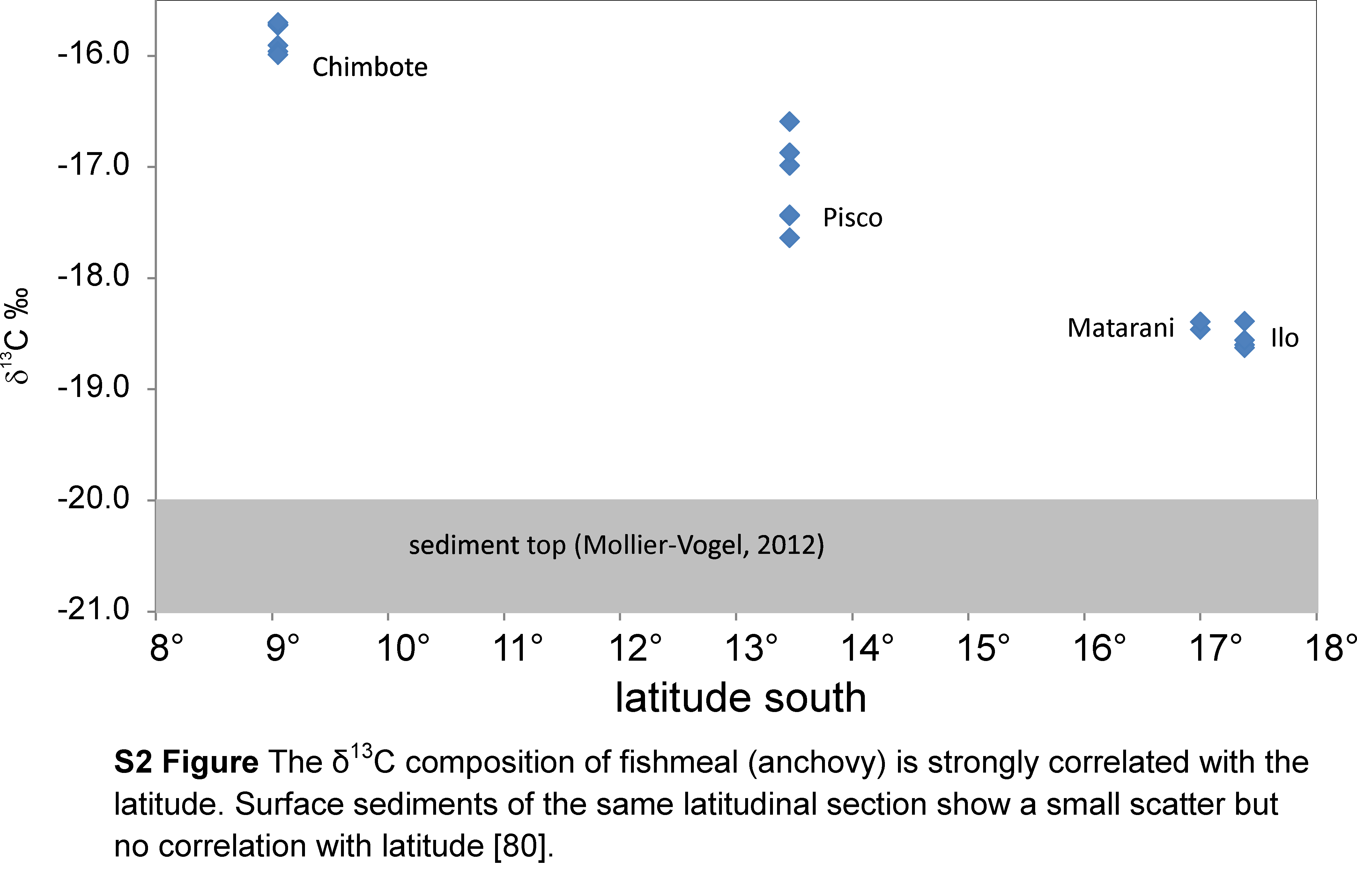

Supplement: S2 Fig — Surface sediments of the same latitudinal section show a small scatter but no correlation with latitude [80]. (TIF) [file pone.0179440.s004.tif]

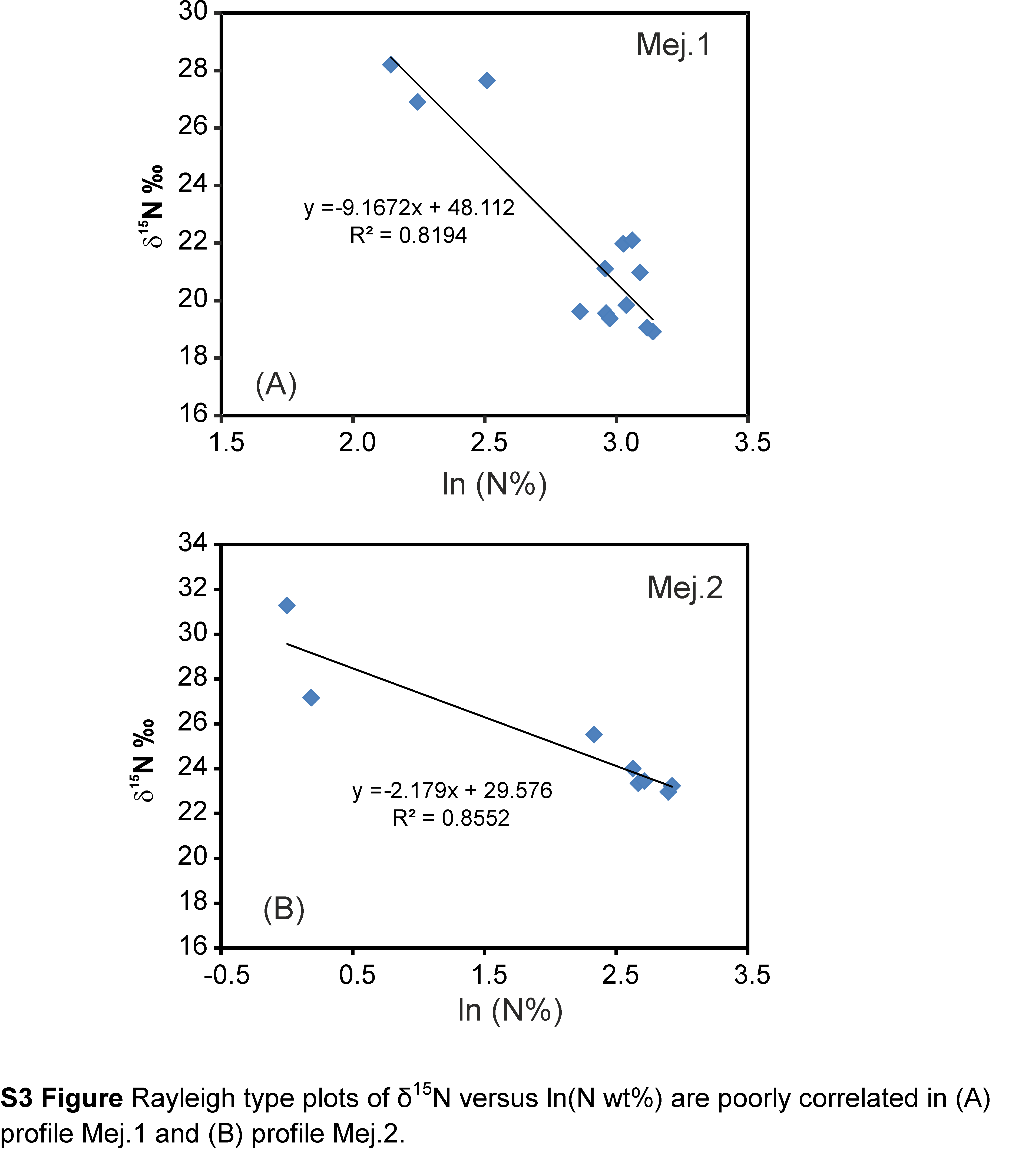

Supplement: S3 Fig — Rayleigh type plots of δ15N versus ln (N wt%) are poorly correlated in (A) profile Mej.1 and (B) profile Mej.2. (TIF) [file pone.0179440.s005.tif]

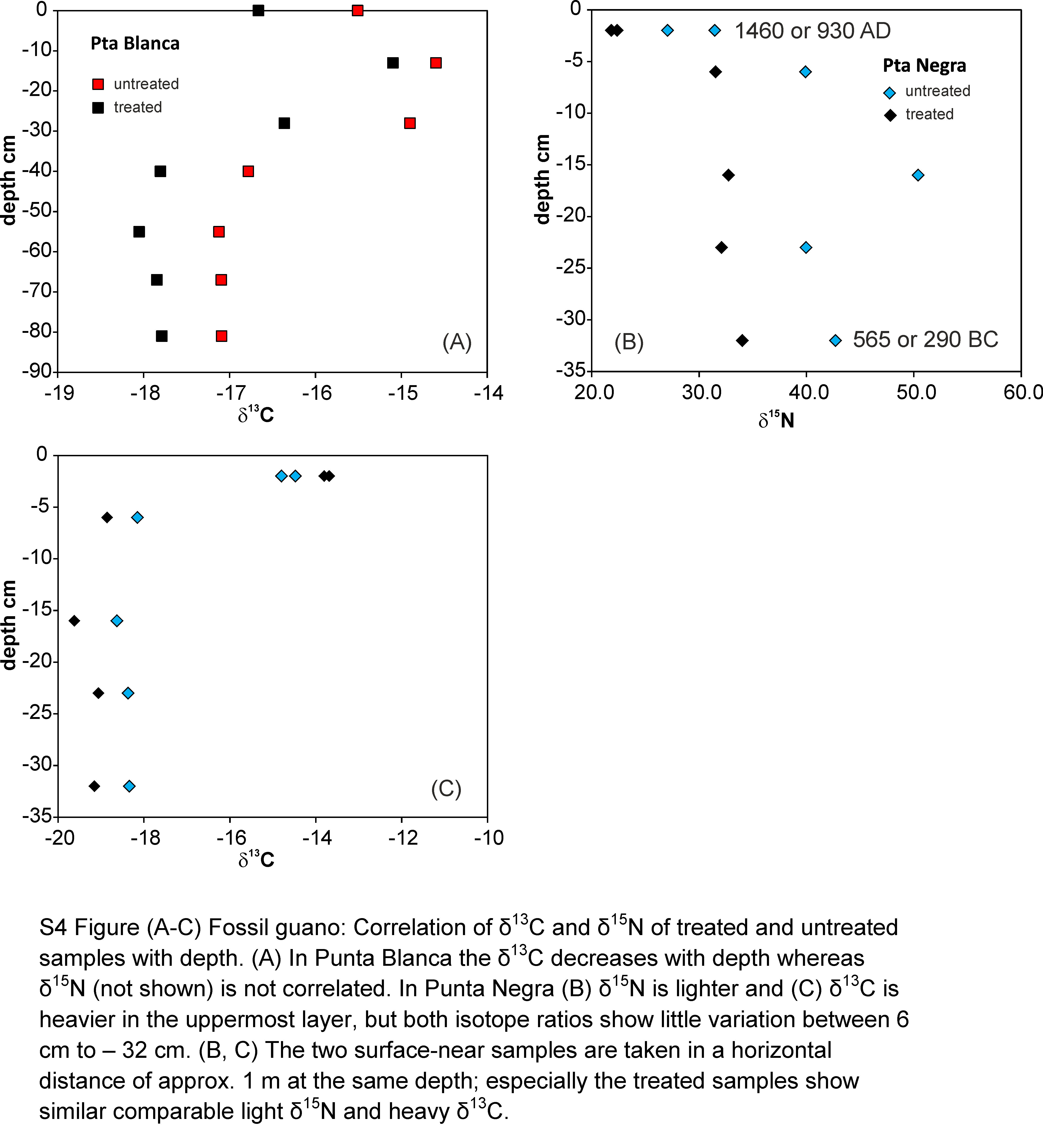

Supplement: S4 Fig — (A-C) Fossil guano: the correlation of δ13C and δ15N of treated and untreated samples with increasing depth. (A) In Punta Blanca the δ13C decreases with increasing depth whereas δ15N (not shown) is not correlated. In Punta Negra (B) δ15N is lighter and (C) δ13C is heavier in the uppermost layer, but both isotope ratios show little variation between -6 cm to –32 cm. (B, C) The two close-to-surface samples are taken in a horizontal distance of 1 m at the same depth; especially the treated samples show similar comparably light δ15N and heavy δ13C. (TIF) [file pone.0179440.s006.tif]
